# Anterior column reconstruction with mono-segmental fusion and lag screw fixation of sagittal split for burst-split fracture of the thoracolumbar spine

**DOI:** 10.1007/s00068-025-03018-y

**Published:** 2025-11-25

**Authors:** Lorin M. Benneker, Vasileios Despotidis, Sebastian Wangler, Marius Johann Baptist Keel, Moritz C. Deml, Jan Gewiess, Christoph E. Albers

**Affiliations:** 1 Orthopädie Sonnenhof, Bern, Switzerland; 2 Department of Orthopaedic Surgery and Traumatology, Bern University Hospital, University of Bern, Bern, Switzerland; 3 Trauma Center Hirslanden, Clinic Hirslanden Zürich, Medical School University of Zürich, Zürich, Switzerland

**Keywords:** Spine, Thoracolumbar, Burst fracture, Surgical technique, Lag screw

## Abstract

**Objectives:**

To present and evaluate the safety and efficacy of our new procedure for treating thoracolumbar burst-split fractures without neurological injury.

**Methods:**

Our new surgical technique for the treatment of thoracolumbar burst-split fractures (AO type A4, Magerl classification A3.2.1) involving (1) posterior reduction and bisegmental instrumention, (2) anterior screw fixation of the caudal sagittal split, (3) anterior one-level fusion of the cranial segment, and (4) interval posterior implant removal was presented. In an initial cohort of patients, demographic information, surgical specifics and imaging data were evaluated.

**Results:**

Twenty-one patients (mean age 29.5 ± 11.8 years, 38% male, mean follow-up 36 ± 14 months) were included. Anterior column reconstruction involving sagittal split lag screw and monosegmental fusion was performed at a mean of 2.9 ± 2 days after posterior instrumentation. All fractures healed. There were no occurrences of implant failures or migrations. None of the patients required revision surgery. The removal of the temporary posterior instrumentation was performed at a mean of 8.4 ± 1.8 months after the initial surgery. Bisegmental, superior monosegmental, and inferior monosegmental kyphosis angle did not significantly change from six months to 12 months postoperatively after removal of the posterior instrumentation (*p* > 0.9). No listhesis or change in bisegmental scoliosis angle were observed. The inferior monosegmental angle was significantly greater in flexion (1.2° ± 5.8°) compared to extension (-3.3° ± 6°) at 12 months postoperatively indicating motion in the inferior, non-fused segment after removal of the posterior instrumentation (*p* = 0.0001). The intervertebral disc height at the temporarily fused segment decreased significantly from six (9.2 ± 2.2) to 12 months postoperatively (8.3 ± 2.2; *p* < 0.0101).

**Conclusion:**

Thoracolumbar burst-split fractures can be safely and successfully treated through a treatment protocol that includes (1) posterior reduction and bisegmental instrumention, (2) anterior screw fixation of the caudal sagittal split, (3) anterior one-level fusion of the cranial segment, and (4) interval posterior implant removal. This new surgical technique promotes reliable fracture healing, kyphosis correction and preserves the physiological motion at the caudal segment.

## Introduction

**Fig. 1:**
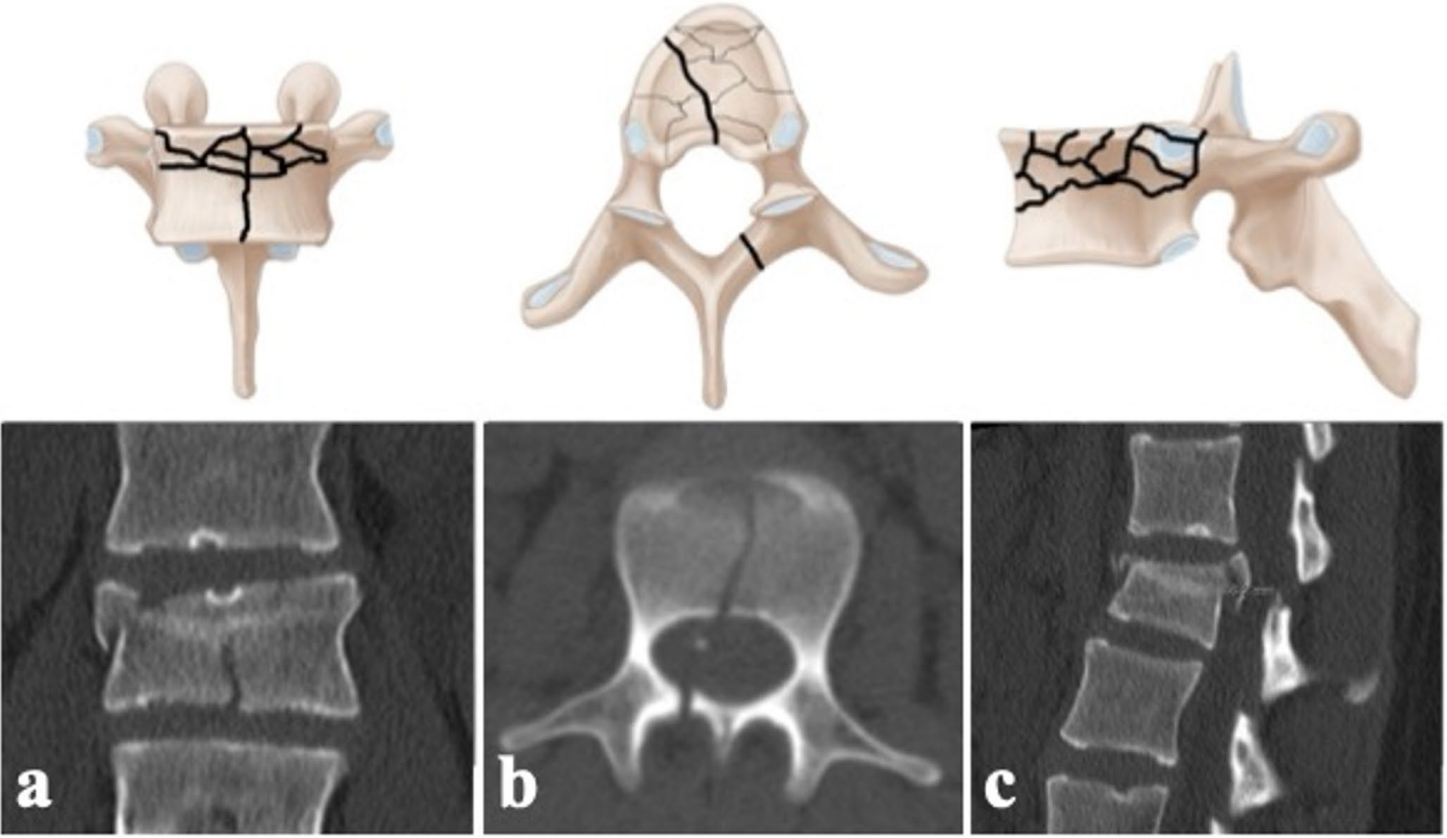
Schemes and CT-scans representing a burst-split fracture (AO Spine A4 type) in (**a**) anteroposterior, (**b**) axial, and (**c**) sagittal views

The fracture pattern of thoracolumbar burst-split fractures is characterized by a fracture of the superior endplate with involvement of the posterior wall and a sagittal split through the inferior endplate typically extending through the posterior vertebral arch (Fig. 1). Consequently, burst-split fractures represent a subgroup of complete burst fractures (AO Spine A4 type [1]; Magerl-classification type A3.2.1 [2]). At present, there is no consensus regarding the treatment of thoracolumbar burst fractures without neurologic deficit and without disruption of the posterior ligamentous complex [3–11].

In our clinical practice, we conduct anterior column reconstruction with two-level fusion for complete burst fractures (AO Spine A4 type), along with percutaneous, two-level posterior instrumentation. The goal of this 360° approach is to achieve long-term stability of the fractured vertebra, restore and maintain sagittal and coronal alignment while preserving as many motion segments as possible. As a result, we often recommend the removal of posterior instrumentation 6 to 12 months after the initial surgery to facilitate the liberation of the caudal motion segment.

**Fig. 2:**
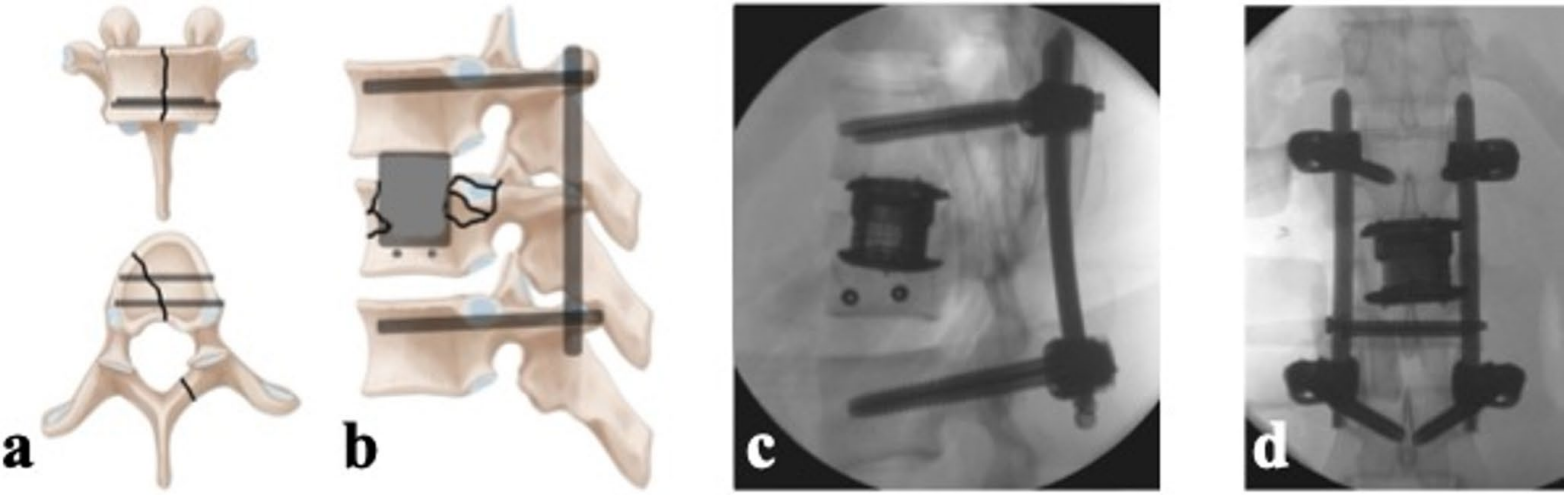
Schemes representing the presented approach with (**a**) sagittal split lag screw fixation and (**b**) mono-segmental anterior fusion with temporary short posterior stabilization. Representative sagittal (**c**) and a.-p. (**d**) intraoperative fluroscopy of a complete burst-split fracture treated using the screw/monosegmental fusion technique

In the subset of burst-split fractures, we consistently observed a widening of the split component during osteotomy or cage expansion with subsequent migration of the cage into the caudal part of the vertebra, loss of correction and posttraumatic disc degeneration. Therefore, we have devised a new surgical technique involving (1) posterior reduction and bisegmental instrumentation, (2) anterior screw fixation of the caudal sagittal split, (3) anterior one-level fusion of the cranial segment, and (4) interval posterior implant removal (Fig. 2). This study aims to evaluate the safety and efficacy of our innovative procedure for treating thoracolumbar burst-split fractures. Furthermore, we will present clinical and radiological outcomes from an initial cohort of patients who underwent treatment with our new surgical technique.

## Methods

We hypothesized that our new surgical technique involving (1) posterior reduction and bisegmental instrumentation, (2) anterior screw fixation of the caudal sagittal split, (3) anterior one-level fusion of the cranial segment, and (4) interval posterior implant removal (Fig. 2) is safe and effective in the treatment of thoracolumbar burst-split fractures.

### Participants

This retrospective observational study was performed in accordance with the guidelines of the Declaration of Helsinki and approved by the local ethics committee. We searched patients suffering thoracolumbar burst-split fractures who underwent stabilization using our new surgical technique between 2012 and 2020. Inclusion criteria mandated a comprehensive 12-month follow-up encompassing both clinical and radiographic assessments. Patients with incomplete clinical or radiological data, loss to follow up, or preoperative neurologic impairment were excluded.

## New surgical technique

Patients with thoracolumbar burst-split fractures often experience high-energy trauma and are admitted to our level I trauma center through the resuscitation room (Table 1). Following assessment according to the Advanced Trauma Life Support (ATLS^®^) guidelines, stabilization, and clearance for surgery, a two-stage procedure is typically performed (Fig. 2). This procedure is indicated, if the extent of comminution would allow safe placement of 3.5 mm screws without breeching into the caudal endplate or cranial burst part of the fracture (less than 60% comminution of the vertebral body, McCormack grade 2 comminution [12]).

**Table 1:** Age, sex, and fracture locations of included patients

Parameter	Value
Age (mean ± SD)	29.5 ± 11.8
Sex (m: f)	8 (38%) : 13 (62%)
ASA score (median, IQR)	1, 2
Trauma mechanism (n (%))	
- motor vehicle accident	5 (24)
- fall from height	7 (33)
- sports accident	9 (43)
Fracture location (n (%))	
- TH12	3 (14)
- L1	9 (43)
- L2	4 (19)
- L3	3 (14)
- L4	2 (10)
Preoperative McCormack score (n (%)) [points]	
− 2	2 (11)
− 3	6 (32)
− 4	3 (16)
− 5	5 (26)
− 6	3 (16)

*ASA* American Society of Anesthesiologists, *IQR* interquartile range, *SD *standard deviation

During the first stage, posterior two-level instrumentation with monoaxial pedicle screws is carried out using a percutaneous or open approach. Decompression may be performed at the surgeon’s discretion in cases of fragment retropulsion in the spinal canal without neurological impairment.

The second stage involves an anterior approach to perform screw osteosynthesis of the caudal sagittal split and hemi-corpectomy with one-level fusion of the cranial burst. Depending on the fracture level, this procedure may be thoracoscopically assisted, performed through a mini-thoracotomy, or via lumbotomy. A SynFrame^®^ retractor (Depuy/Synthes) attached to the surgical table is commonly used to provide a comprehensive exposure. The injured vertebral body is identified, adjacent disc spaces are exposed, and correct level verification is done fluoroscopically. Segment vessels are cauterized or ligated as needed. Two drill bits are inserted across the caudal sagittal split, with fluoroscopic confirmation of correct positioning and length in both anteroposterior and lateral views. Subsequently, fully threaded 3.5 mm cortical screws are inserted using a lag screw technique to achieve interfragmentary compression after removal of the drill bits. Alternatively, two partially threaded screws may be utilized based on the surgeons’ preference. In the region of the cranial burst fracture, an incomplete, superior corpectomy is performed along with the removal of the superior adjacent disc. The excised bone is harvested and utilized as a graft material to fill the cage. Following the placement of a trial cage for assessing correct implant sizing and fit, the final cage (Stryker VLIFT ^®^; WORC RecoLift; DePuy Synthes Synex™) is implanted.

Depending on accompanying injuries, the postoperative mobilization protocol involves full weight-bearing with caution against excessive movement. No corset or orthosis is prescribed. Non-contact sports are allowed from three months postoperatively, and contact and high-impact sports are permitted from six months postoperatively.

Following the regular six month postoperative follow up visit, all patients undergo CT imaging to assess fracture consolidation and the osseous integration of the cage. If satisfactory cage integration and fracture healing are observed, removal of the posterior pedicle screw instrumentation is recommended for the liberation of the caudal, non-fused motion segment.

### Endpoints and objectives

#### Primary outcomes

The primary objective of this study was to document the feasibility, clinical outcomes, and potential complications associated with the specified surgical procedure. Data pertaining to patient demographics (including age, BMI, mechanism of trauma, symptoms, gender, preexisting medical conditions, and hospitalization details) were extracted from medical records. Furthermore, surgical specifics (such as approach, duration, types of implants utilized, amount of blood loss, and any complications) were obtained from the patients’ medical charts. CT scans obtained post-trauma and prior to the removal of temporary stabilization were analyzed to assess the fracture characteristics and the size of the sagittal split by measuring the largest gap diameter on coronal CT scans, integration of the cage and the healing of the inferior sagittal split. Clinical outcomes were evaluated using the numeric rating scale (NRS) to gauge pain levels at 6 and 12 months following the surgical procedure.

#### Secondary outcomes

**Fig. 3:**
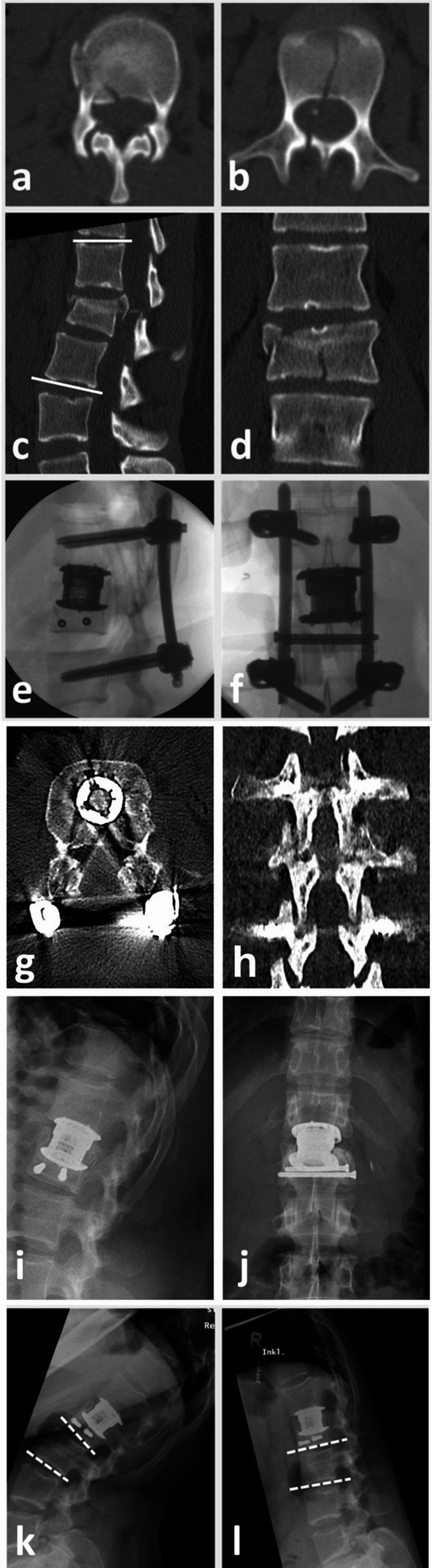
Representative case of a 30-year-old female patient suffering a burst-split fracture of L2 (AO Spine L1-2: B2 (L2: A4; N2; M0)) following a car accident (**a**-**d**). Axial reconstruction shows the burst fracture in the cranial region (**a**) and the split fracture in the caudal region (**b**) of the vertebral body. The sagittal split size was quantified by measuring the largest gap diameter on coronal CT scans after trauma (**d**). The patient underwent posterior instrumentation of L1-L3 with monosegmental spondylodesis at the L1/2 level, decompression with clearance of the spinal canal, and sagittal split screw fixation and monosegmental anterior fusion at L1/2 via lumbotomy (**e**, **f**). CT imaging after six months showed fracture consolidation of the caudal split, osseous integration of the monosegmental cage, and fusion of the posterior elements (**g**, **h**). The patient was scheduled for removal of the posterior instrumentation (**i**, **j**). To characterize the inferior intervertebral discs' functionality after removal of the dorsal stabilization, segmental range of motion was assessed comparing the intervertebral disc angle (dashed lines) in flexion (**k**) and extension (**l**) lateral standing radiography at 12 months after the injury

The secondary objective of this study was to document the radiological outcomes following the described procedure. Radiological parameters were evaluated during regular postoperative follow-up appointments at our institution at six and 12 months postoperatively. Measurements were conducted on Digital Imaging and Communications in Medicine (DICOM) formatted images (Sectra IDS7™ system). Fracture kyphosis was assessed on lateral standing radiographs. Bisegmental kyphosis was determined utilizing the Cobb measurement technique from the superior endplate of the adjacent cranial vertebral body to the inferior endplate of the adjacent caudal body (bisegmental angle). Additionally, the superior and inferior kyphosis angles were calculated based on the superior endplate of the vertebral body above and the inferior endplate of the fractured vertebral body (superior monosegmental angle), as well as the inferior endplate of the fractured vertebral body and the inferior endplate of the vertebral body below (inferior monosegmental angle). The segmental scoliosis angle was assessed using Cobb’s method on anterior-posterior standing radiographs. To evaluate the functionality of the inferior intervertebral discs after dorsal stabilization removal, segmental range of motion was measured using the intervertebral disc angle (Fig. 3) during flexion/extension lateral standing radiography. Furthermore, listhesis at the temporarily stabilized segment was evaluated on lateral standing radiographs in upright, flexion, and extension positions at 12 months postoperatively and graded according to the Meyerding classification. The height of the intervertebral disc below the fractured vertebra was determined on lateral standing radiographs following the method described by Frobin et al. (anterior disc height divided by the mean depth of the cranial vertebral body) [13].

#### Statistical analysis

Statistical evaluation was performed using GraphPad Prism (Version 10.2.0 (2024); GraphPad Software, La Jolla, CA, USA). Normal distribution was assessed according to the Shapiro Wilk test. Descriptive statistics are reported with means and standard deviations (SD) and median and interquartile range (IQR) as well as minimum and maximum values, respectively. For paired analysis of grouped data, the paired t test and Wilcoxon matched-pairs signed rank test were performed. The level of significance was set at *p* = 0.05.

## Results

### Participants

We identified 32 patients who underwent intentional treatment utilizing our new technique. Of these, eleven patients were excluded from the study: Two due to initial neurological impairment, three due to incomplete radiological follow-up, one due to death unrelated to the procedure, and five due to loss to follow-up. The final analysis included 21 patients who met the inclusion criteria, with a mean age of 29.5 ± 11.8 years (range: 17 to 65). Among the included patients, eight (38%) were male. The average follow-up period was 36 ± 14 months (ranging from 21 to 75 months) post the initial operation. Additional demographic characteristics can be found in Table 1.

### Primary outcomes

Twenty of 21 patients (95%) of the patients underwent a staged procedure, with posterior stabilization carried out on the day of injury. The subsequent anterior completion involving sagittal split lag screw and monosegmental fusion was performed at a mean of 2.9 days ± 2 days (ranging from 0 to 7 days) following the posterior procedure. Pre-removal CT scans of the temporary posterior instrumentation revealed monosegmental fusion and/or osseous integration of the cage in all 21 patients. The average sagittal split size was 1.4 mm ± 0.3 mm (ranging from 1 mm to 2 mm) on posttraumatic CT scans. Prior to the removal of temporary stabilization, CT scans demonstrated fracture healing in all cases. The removal of the temporary posterior instrumentation took place at a mean of 8.4 months ± 1.8 months (range 5 to 11 months) after the initial surgery. Additional details regarding surgery-related data can be found in Table 2.

**Table 2:** Data related to surgery of included patients

Parameter	Value
Fracture Level	
Mean interval posterior - anterior (SD, range) [days]	2.9 (2, 0–7)
Mean interval posterior - removal of posterior instrumentation (SD, range) [months]	8.4 (1.8, 5–11)
Type of posterior surgery (n (%))	
- percutaneous	6 (29)
- open with decompression	15 (71)
- index level screw placement	3 (14)
Type of anterior surgery (n (%))	
- lumbotomy	9 (43)
- mini-thoracotomy	10 (49)
- thoracoscopy	2 (10)
Mean duration of surgery (SD) [min]	
- percutaneous posterior instrumentation	73 (24)
- open posterior instrumentation and decompression	115 (15)
- anterior split screw/monosegmental fusion	120 (28)
Blood loss (mean (SD)) [ml]	
- posterior instrumentation	265 (138)
- anterior split screw/monosegmental fusion	339 (204)

*SD* standard deviation

**Fig. 4:**
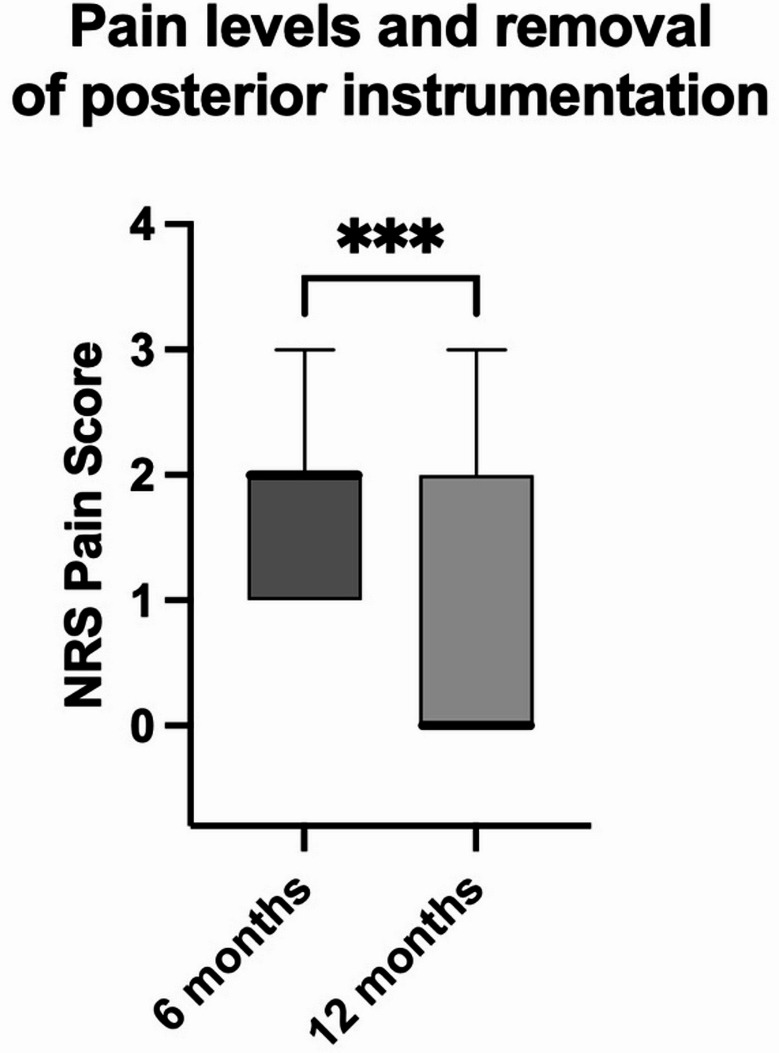
Boxplots of Numeric Rating Scale (NRS) pain levels showing a significant decrease from six to twelve months postoperatively. Line – median. Whiskers – min to max, *** - *p* < 0.001

Six months postoperatively, NRS pain levels were low (median 2, IQR 1). At 12 months postoperatively, there was a significant further decrease of NRS pain levels (median 0, IQR 1.5; *p* = 0.0001, Fig. 4).

A single intraoperative dural tear was noted during posterior decompression at the L1 level and instrumentation of Th12-L2 in one patient. The tear was promptly addressed through dural suturing, and the patient did not exhibit any postoperative clinical symptoms. No additional complications were encountered during the posterior procedure. We observed no intra- or postoperative complications related to the anterior procedure in any patient. There were no instances of new-onset neurologic impairment observed in any patient. Throughout the follow-up period, there were no occurrences of implant failures or migrations. Notably, none of the patients required revision surgery.

### Secondary outcomes

Bisegmental, superior monosegmental, and inferior monosegmental kyphosis angle did not significantly change from six months to 12 months postoperatively (*p* > 0.9; Table 3). Segmental scoliosis did not change significantly over time (*p* > 0.1; Table 3).

**Table 3:** Bisegmental, superior and inferior segmental kyphosis, segmental scoliosis, and disc height of included patients

Parameter	6 months*	12 months	*n* values	*p* value
Bisegmental kyphosis (median, IQR) [°]	10 (14.5)	11.6 (19.9)	19	0.9487
Superior segmental kyphosis (median, IQR) [°]	8.6 (14.7)	10.1 (19)	18	0.9445
Inferior segmental kyphosis (median, IQR) [°]	6.7 (7.4)	5.3 (1.6)	17	0.9008
Segmental scoliosis (median, IQR) [°]	1.4 (2.3)	1.5 (2.5)	19	0.1324
Disc height (median, IQR)	9.2 (2.2)	8.2 (2.2)	20	0.0101

* Prior to hardware removal, *IQR* interquartile range

**Fig. 5:**
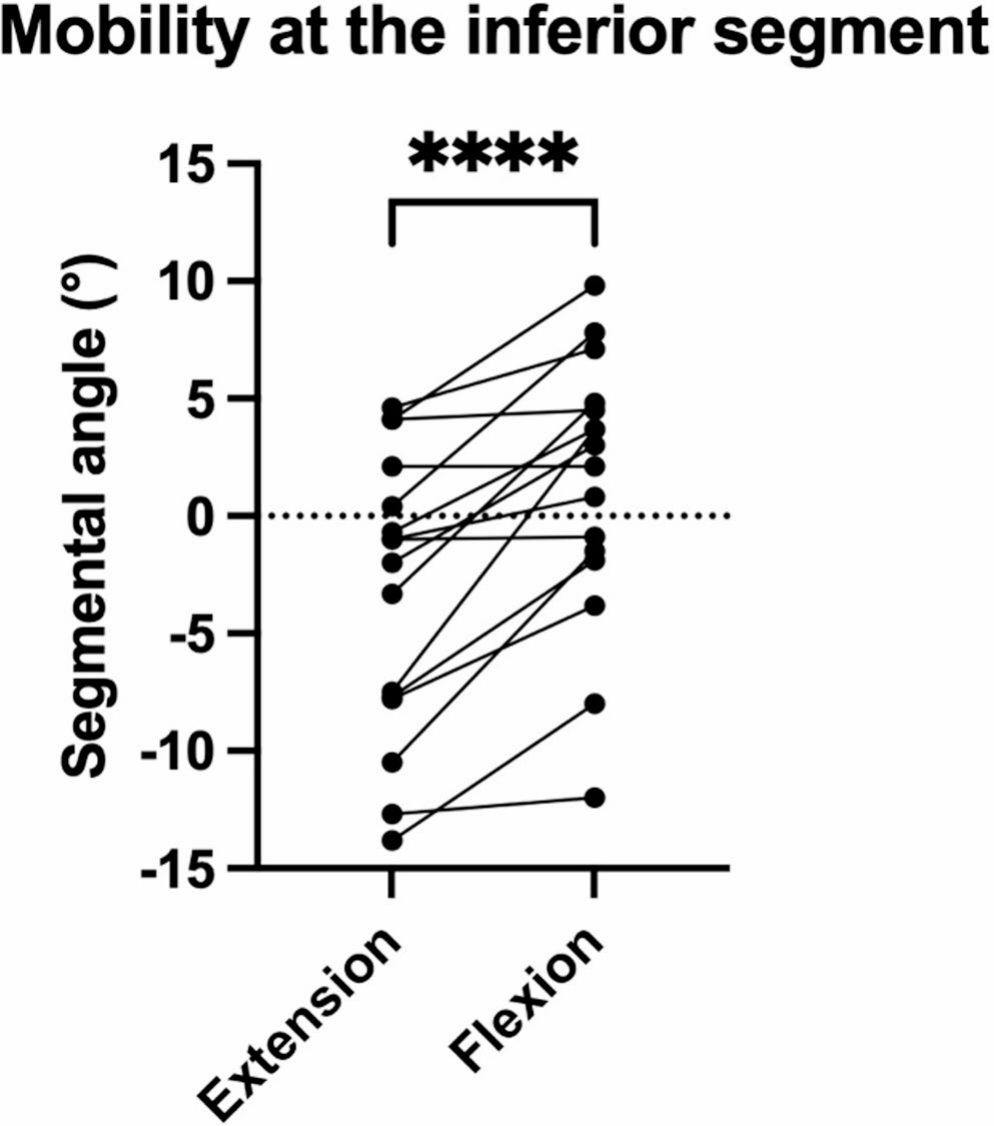
Segmental range of motion assessed comparing the intervertebral disc angle of the non-fused inferior segment in flexion and extension. Compared to extension, the segmental angle was significantly greater in flexion at 12 months postoperatively. Symbol – individual value; line – connecting individual values; **** - *p* < 0.0001

There was a significant difference regarding the intervertebral disc angle of the non-fused inferior segment in flexion (1.2° ± 5.8°, range: −12° to 9.8°) and extension (−3.3° ± 6°, −13.8° to 4.6°) lateral standing radiographs at 12 months postoperatively (*p* = 0.0001; Fig. 5). When comparing the respective fracture levels, the inferior disc mobility was greatest at the L2/3 and L3/4 levels after L2 and L3 fracture treatment, respectively, and smallest at the L4/5 level after L4 fracture treatment (Table 4).

**Table 4:** Level specific intervertebral disc angles in inclination and reclination at 12 months postoperatively

Level	*n* (%)	Disc angle in Flexion (SD) [°]	Disc angle in Extension (SD) [°]	Delta (SD) [°]
TH12/L1	3 (14)	7.1 (2.7)	4.3 (0.3)	2.9 (2.7)
L1/2	9 (43)	1.4 (2.8)	−3.3 (4.2)	4.7 (3.9)
L2/3	4 (19)	4.2 (2.5)	−1.8 (4.2)	5.9 (4.7)
L3/4	3 (14)	−5 (4.3)	−10.8 (4.3)	5.8 (0)
L4/5	2 (10)	−7.9 (5.8)	−10.3 (3.5)	2.4 (2.3)

*SD* standard deviation

We did not observe any new listhesis at the temporarily fused segment in any of the included patients. No evidence of segmental instability on flexion and extension images was noticed.

**Fig. 6:**
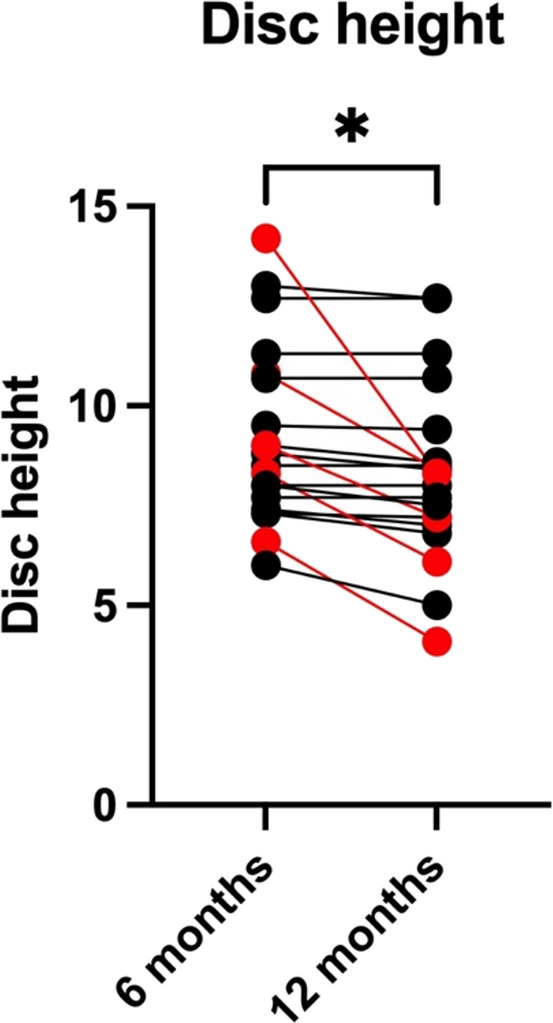
Inferior disc height at 6 months and 12 months postoperatively. There was a significant decrease over time. However, only 5 patients showed a marked decrease in disc height > 1 (red dots/lines). * - *p* < 0.05

The intervertebral disc height at the temporarily fused segment decreased significantly from six (9.2 ± 2.2, ranging from 6 to 8.2) to 12 months postoperatively (8.3 ± 2.2, 4.1 to 12.7; *p* < 0.0101; Table 3; Fig. 6).

## Discussion

The management of thoracolumbar burst fractures remains a contentious issue in spinal traumatology. In this study, we introduced a novel, safe, and feasible approach for treating thoracolumbar burst-split fractures (AO Spine A4 [1], Magerl A3.2.1 [2]). This approach involves (1) posterior reduction and bisegmental instrumention, (2) anterior screw fixation of the caudal sagittal split, 3), anterior mono-segmental fusion of the cranial segment, and 4) interval posterior implant removal with caudal motion segment liberation. Our initial case series demonstrated the safety and efficacy of this technique, showing reliable fracture healing and favorable clinical and radiological outcomes. In essence, this innovative approach contributes to the preservation of motion at the caudal segment.

According to the updated AO Spine classification system, relevant thoracolumbar compression fractures can be categorized as split fractures (A2 type), incomplete burst fractures (A3 type), and complete burst fractures (A4 type) [1]. The characteristic pattern of burst-split fractures typically arises from excessive axial loading of the spine. This results in comminution of the cranial vertebral body portion affecting the superior endplate and posterior wall, along with a sagittally oriented split component of the caudal portion extending through the caudal endplate and posterior lamina [14]. Therefore, thoracolumbar burst-split fractures represent a common subset of complete burst fractures (AO Spine A4 type [1], formerly identified as Magerl A3.2.1 variant [2]).

The efficacy of non-surgical versus surgical interventions for thoracolumbar burst fractures remains a topic of ongoing debate. A recent survey conducted among international spine surgery experts indicated that the majority of surgeons opt for conservative management of incomplete burst fractures (AO Spine A3 type) and surgical treatment of complete burst fractures (AO Spine A4 type) [15]. However, a Cochrane review highlighted contradictory evidence from two trials, leading to inconclusive findings on the pain and functional outcomes associated with conservative versus operative management of complete burst fractures [3]. In our clinical approach, conservative management of thoracolumbar burst fractures is considered appropriate only for patients deemed unfit for surgical intervention. Kyphoplasty and stent-kyphoplasty have shown positive clinical and radiographic outcomes based on short-term follow-up data, but they are associated with a notable rate of pseudarthroses and secondary loss of reduction [16–18]. In comparison to kyphoplasty, posterior instrumentation offers superior alignment reduction [19]. Several meta-analyses examining posterior-only, anterior-only, and anteroposterior stabilization approaches over the past decade have indicated that posterior-only instrumentation is linked to reduced intraoperative blood loss and shorter surgical duration. However, it is also associated with higher VAS pain scores, while rates of implant failure, infection, and revision surgery were similar [20–22]. Notably, posterior-only instrumentation carries the potential risk of alignment loss and progressive kyphosis following implant removal [23–25].

Screw osteosynthesis is a well-established technique in managing long bone fractures. Yet, lag screws have not been traditionally utilized in spinal fracture treatment. Our clinical observations revealed challenges when attempting to preserve the caudal disc by replacing only the cranial disc and superior vertebra, and performing a one-level fusion in burst-split fractures, resulting in frequent failures. One notable failure mode involved intraoperative displacement of the split caudal vertebra while expanding the cage. The extent of split displacement during trauma may be underestimated in imaging studies, and the iatrogenic repetition of this phenomenon carries the potential risk of significant spinal canal compression [14]. To address these issues, our proposed technique involves stabilizing the split component using two lateral lag screws to enhance stability of the lower hemivertebra, (e.g., converting a type A4 fracture into a type A3 fracture), thereby facilitating subsequent anterior column reconstruction with one-level fusion. As demonstrated in our initial case series, implementation of this novel approach successfully averted the previously observed failure mode and did not result in any other surgery-related complications. A biomechanical analysis of lag screw osteosynthesis in vertebral split fractures demonstrated effective approximation of fragments, elimination of fracture gaps, and promotion of bony fragment contact essential for fracture healing [26]. These findings were corroborated by our postoperative CT assessments, revealing complete healing of all fractures six months after split screw implantation, absence of relevant fracture gaps, and no occurrence of pseudarthrosis at the split or cranial fusion site.

Another observed failure mode was the accelerated degeneration of the non-fused caudal disc. Vertebral fractures involving the endplates are recognized to trigger degenerative alterations in the intervertebral disc [27]. Research has demonstrated that vertebral endplate trauma can lead to disc cell apoptosis and contribute to organ degeneration in vitro [28]. Moreover, post-fracture vertebral endplate sclerosis may hinder the nutritional supply to intervertebral disc cells, which is believed to be a contributing factor in disc degeneration [29]. Altered biomechanics, such as sustained increased mechanical loading on the disc, can induce degenerative changes and result in low back pain [30].

The removal of posterior instrumentation implants may help prevent future adjacent segment disease, while the advancement of kyphosis can be effectively halted by anterior column reconstruction [31]. We believe that the benefits of the new surgical technique clearly outweigh the drawbacks of multi-segment posterior instrumentation, which carries risks of compromising long-term function, progressive kyphosis post-implant removal, and adjacent segment disease. Our findings suggest that temporary short-segment posterior instrumentation can preserve the functionality of the caudal disc at 1 year follow-up. The removal of implants and liberation of the caudal segment may also impact perceived pain levels, as indicated by our results. However, a significant reduction in disc height was observed following the liberation of the caudal segment. This change in disc height may be partially attributed to the screw configuration, where monoaxial screws prevent alterations in disc height, while initial posterior instrumentation and fracture reduction may cause some distraction leading to a change in disc height compared to the pre-injury state.

Our case series study has several limitations that warrant acknowledgment. Firstly, the retrospective study design inherently introduces biases and constraints in data collection and analysis. Exclusion of patients with incomplete data or loss to follow-up may have introduced selection bias. Furthermore, functional outcomes were limited to pain scores, as patient-reported outcome measures were not consistently collected. Their inclusion would strengthen the clinical impact of this study. Although MRI is the gold standard to assess disc degeneration we had to choose an indirect assessment with radiographic parameters including motion. The lack of routine preoperative and postoperative MRI scans can be attributed to their favorable clinical status, which obviates the necessity for postoperative imaging. Additionally, the study was based on a small case series. However, the demographic characteristics of the patients included in this study suggest that the study population and fracture locations align with those typically observed in cases of traumatic thoracolumbar fractures. The relatively high rate of loss to follow-up can be attributed in part to foreign patients receiving subsequent care and follow-up in their home countries. The absence of a control group limits the ability to establish comparative efficacy. Future research will aim to compare this technique with nonoperative management and posterior-only fixation. Potential concomitant injuries were not taken into account, although neurological deficits were ruled out based on the inclusion criteria. The 36-month mean follow-up period may be insufficient to draw definitive conclusions on the incidence and risk of long-term complications such as adjacent segment disease. Nonetheless, no patients treated with our new surgical technique presented with symptomatic adjacent segment disease requiring revision surgery during the follow-up period. Our cohort was relatively young. The applicability of this technique in older adults may be restricted due to bone quality and comorbidities.

## Conclusion

Thoracolumbar burst-split fractures can be successfully treated through a treatment protocol that includes (1) posterior reduction and bisegmental instrumention, (2) anterior screw fixation of the caudal sagittal split, (3) anterior one-level fusion of the cranial segment, and (4) interval posterior implant removal. This new surgical technique represents a safe and feasible treatment strategy that promotes reliable fracture healing and leads to favorable clinical and radiological outcomes. Additionally, this innovative approach contributes to the preservation of motion at the caudal segment. Prospective, long-term studies involving larger cohorts with control groups and serial MRI follow-up are warranted to augment the findings of the current study.

## Data Availability

No datasets were generated or analysed during the current study.
